# When formal governance persists but resilience does not: health system governance and resilience in Yemen across a decade of conflict and crisis (2014–2025)

**DOI:** 10.1186/s12913-026-14957-6

**Published:** 2026-06-18

**Authors:** Dalia Hyzam, Michael Boah, Mustafa Dhaiban, Fekri Dureab, Taha Al-Mahbashi, Aiman Hadi, Khaled Al Sakkaf, Gaby Feldmann, Timo Ulrichs

**Affiliations:** 1 Institute for Research in International Assistance (IRIA), Akkon University, Pasewalker Straße 112, 13127 Berlin, Germany; 2 Centre for Population Health, University of Global Health Equity (UGHE), Kigali, Rwanda; 3 Department of Public Health, College of Medicine and Health Sciences, Shabwah University, Shabwah, Yemen; 4 Department of Healthcare Management, Collage of Business, University of Doha for Science and Technology, Doha, Qatar; 5 Faculty of Medicine and Health Sciences, Al-Razi University, Sana’a, Yemen; 6 College for Health Sciences, National University College for Health Sciences, Sana’a, Yemen; 7 Center for Global Health and International Development (GHID), Freiburg International Academy (FIA), Freiburg, Germany; 8 Charité – Universitätsmedizin, Berlin, Germany; 9 Department of Community Medicine, Public Health Faculty of Medicine and Health Sciences, University of Aden, Aden, Yemen

**Keywords:** Adaptive capacity, Fragile and conflict-affected settings, Health systems, Resilience, Governance, Yemen

## Abstract

**Background:**

Yemen’s health system has faced prolonged shocks, including war, epidemics, and COVID-19, challenging both governance and resilience. This study examines how formal governance structures and institutional capacities shaped the system’s ability to anticipate, absorb, adapt, learn, and transform across a decade of crisis (2014–2025).

**Methods:**

We conducted a longitudinal qualitative analysis based on interviews with health system leaders and technical actors. Using a resilience-capacity framework, we analyzed governance functions across four phases: pre-conflict period (pre-2014), conflict escalation (2015–2019), COVID-19, and the post-pandemic period. Data were thematically coded and interpreted through the sequential resilience lens of preparedness, absorption, adaptation, learning, and transformation.

**Results:**

Participants consistently described a health system with intact formal governance structures but fragile functional capacity. Pre-2014 governance was administratively stable yet centralized and poorly prepared for shocks. During the conflict, preparedness collapsed amid fragmented authorities, and absorptive capacity relied heavily on donor-driven service delivery. COVID-19 triggered temporary improvements in coordination and emergency response, but these were largely ad hoc and poorly institutionalized. Post-pandemic, preparedness remained procedural, absorptive capacity weakened as external funding declined, adaptive measures persisted in localized and reversible forms, and transformative governance remained constrained by political instability and weak enforcement. Digital “workaround governance,” including WhatsApp-based coordination, facilitated rapid decision-making but highlighted gaps in formal systems and accountability. Across phases, learning was fragmented, donor-driven, and rarely institutionalized, limiting system-wide reform.

**Conclusion:**

Yemen’s experience demonstrates that maintaining formal governance structures does not guarantee health system resilience. Sustainable resilience requires institutionalized preparedness, domestic contingency financing, integration of adaptive innovations, and embedding learning into routine governance. Efforts to strengthen health systems in fragile and conflict-affected settings must address governance, capacity, and political economy simultaneously to move beyond reactive crisis management toward transformative change.

**Supplementary Information:**

The online version contains supplementary material available at 10.1186/s12913-026-14957-6.

## Introduction

Health systems operating in fragile and conflict-affected settings are routinely exposed to compounded shocks that test their capacity to sustain essential functions, protect population health, and adapt under conditions of extreme uncertainty. Over the past two decades, protracted armed conflicts, political fragmentation, economic collapse, and recurrent public health emergencies have increasingly converged, underscoring the importance of health system resilience as both an analytical and operational priority [1]. Resilience, in this context, extends beyond short-term crisis response to encompass the ability of health systems to anticipate, absorb, adapt to, and transform in response to sustained stressors while maintaining core functions and equity-oriented outcomes [2].

Yemen represents one of the most severe contemporary examples of health system fragility. Prior to 2014, the Yemeni health system was already characterized by structural weaknesses, including chronic underinvestment, heavy reliance on out-of-pocket payments, workforce shortages, and uneven service coverage between urban and rural areas [3–5]. Governance arrangements were centralized but institutionally weak, with limited regulatory enforcement, fragmented planning, and constrained accountability mechanisms. While basic health services were largely functional, system performance remained vulnerable to political instability and economic shocks [3].

The escalation of armed conflict in 2015 marked a critical rupture in Yemen’s health system trajectory. Widespread destruction of infrastructure, displacement of health workers, salary interruptions, and institutional fragmentation severely undermined service delivery and stewardship functions [4]. The health system became increasingly dependent on humanitarian financing and externally driven vertical programs, leading to parallel coordination structures and further erosion of national leadership and ownership [6, 7]. These dynamics were compounded by macroeconomic collapse, restrictions on imports, and the politicization of aid, which collectively constrained both operational capacity and strategic decision-making.

Subsequent shocks, including recurrent disease outbreaks and the COVID-19 pandemic, further exposed the limitations of Yemen’s health system resilience. While emergency responses were mounted, they were often reactive, short-term, and heavily donor-led, with limited integration into national systems or learning mechanisms [7, 8]. Surveillance, preparedness planning, and institutional memory remained weak, and opportunities for system reform or transformation were frequently deferred due to insecurity and political fragmentation. As a result, the health system has continued to operate in a state of chronic crisis, with resilience manifested primarily as coping rather than sustained adaptation or transformation.

Recent global health discourse has emphasized the central role of governance and leadership in shaping health system resilience, particularly in fragile settings [2, 9, 10]. Effective stewardship, coordination, legitimacy, and learning are increasingly recognized as critical determinants of how systems respond to repeated shocks [11]. However, empirical evidence from protracted conflict contexts remains limited, and much of the existing literature relies on secondary data, humanitarian perspectives, or short-term crisis assessments [12]. There is a relative paucity of longitudinal, qualitative research capturing the perspectives of national and sub-national policymakers who have navigated health system governance across different phases of crisis.

Understanding how health leaders and decision-makers in Yemen have perceived, managed, and adapted governance and leadership functions over time is essential for advancing both theory and practice on health system resilience [13]. Examining experiences across the pre-conflict period, active conflict, the COVID-19 pandemic, and the current protracted crisis provides a unique opportunity to explore how institutional arrangements, power dynamics, and external influences have shaped system performance and resilience capacities over more than a decade.

This study examines the perspectives of senior health policymakers and system leaders on health system performance, governance, and resilience in Yemen between 2014 and 2025. Guided by the World Health Organization (WHO) health system building blocks and a health system resilience framework, it analyses how governance and leadership arrangements have shaped the system’s capacity to respond to repeated shocks.

## Methodology

### Study setting

The study was conducted in Yemen, a fragile and conflict-affected setting characterized by prolonged political instability, economic collapse, and humanitarian crisis [14]. Prior to the escalation of conflict in 2015, Yemen’s health system was characterized by a predominantly public-sector delivery model with centralized governance, limited fiscal space, and persistent structural weaknesses, including underinvestment, fragmented service delivery, and uneven access to care across governorates [15]. Since 2015, the health system has experienced severe institutional fragmentation, donor dependency, workforce attrition, and geographical inequities in service availability and governance capacity [4]. Given Yemen’s institutional fragmentation, this analysis focuses primarily on the formal health system under the internationally recognized Government of Yemen and the Ministry of Public Health and Population in Aden, while acknowledging that health governance in Yemen remains territorially and politically divided. Participants were selected because of their senior roles in national and sub-national health institutions and their experience in policy formulation, donor coordination, emergency response, and system planning. Many had professional experience spanning both the pre-2014 period and subsequent crisis phases, which enabled longitudinal reflection on changes in governance and resilience over time.

Despite these challenges, the health system has continued to function through a combination of humanitarian support, localized adaptation, and informal governance arrangements. However, this survival has occurred in the absence of stable financing, coherent national stewardship, or institutionalized mechanisms for preparedness, learning, and reform, providing a critical context for examining how governance and leadership shape health system resilience under conditions of prolonged conflict, political fragmentation, and recurrent public health shocks.

### Study design and conceptual framework

This study adopted a qualitative exploratory design to examine health system performance and resilience in Yemen across multiple phases of shock: the pre-conflict period (pre-2014), the active conflict phase (2015–2019), the COVID-19 pandemic, and the current protracted crisis context (2020–2025). A qualitative approach was selected to capture policymakers’ experiences, institutional processes, and governance dynamics that are not readily observable through quantitative methods [16].

The study was guided by two complementary analytical frameworks: (1) the WHO health system building blocks framework and (2) a health system resilience lens focusing on preparedness, absorption, adaptation, learning/recovery, and transformation capacities [17]. The WHO health system building blocks were not used as a governance framework. Rather, they were used to structure the inquiry across major health system components affected by shocks, including leadership and governance, financing, workforce, health information systems, medical products and technologies, and service delivery. Governance was analyzed as a cross-cutting function operating through authority, coordination, accountability, decision-making, information flows, and institutional learning across these components. The resilience-capacity lens was then used to interpret whether governance arrangements enabled preparedness, absorption, adaptation, learning/recovery, or transformation across the four study periods.

This combined framework enabled an examination of both system components and cross-cutting governance and resilience processes over time.

In this study, we use health system performance to refer to the functioning of the health system before major shocks, particularly the pre-2014 period. We use health system governance to refer to the arrangements through which authority, coordination, accountability, decision-making, information use, and institutional learning are exercised across the health system. We use health system resilience to refer to the capacity of the system to prepare for, absorb, adapt to, learn from, and transform in response to repeated shocks. The study therefore examines how health system governance and leadership arrangements shaped resilience capacities across successive crisis periods in Yemen.

### Study population and sampling

Purposive sampling was used to recruit senior health policymakers and leaders with direct involvement in health system governance and crisis response. Thirty in-depth interviews were conducted with participants from national and sub-national levels, including directors-general within the four sectors of the Ministry of Public Health and Population (MoPHP), heads of key technical departments, and senior advisors engaged in policy formulation, donor coordination, and system planning.

Participants were selected based on their institutional role, length of experience spanning pre- and post-2015 periods, and involvement in major public health emergencies, including COVID-19. Sampling continued until thematic saturation was achieved.

### Data collection

Data were collected through semi-structured in-depth interviews conducted between May and July 2025. The Interview guide was developed in English, translated into Arabic, and iteratively refined. Key domains included governance and leadership, health financing and donor coordination, workforce dynamics, health information systems, emergency preparedness and response, and institutional learning (See supplementary material 1). The interview guide was developed for a broader health system resilience project. The present paper draws specifically on the governance and leadership domains and on governance-related evidence emerging across financing, workforce, information systems, service delivery, preparedness, and institutional learning. This approach was necessary because governance in fragile health systems is not confined to the leadership block alone but is expressed through decision rights, coordination mechanisms, resource allocation, information use, and accountability across the system.

The interview guide was pilot-tested through two preliminary interviews with Yemeni health system experts for clarity. A two-day training was conducted for six data collectors, who conducted the interviews.

All interviews were conducted in Arabic, audio-recorded with consent, and supplemented by field notes. Interviews lasted 60–120 min, allowing for detailed reflection on temporal changes across crisis phases. Interviews were conducted at participants’ workplaces, including the MoPHP, the Faculty of Medicine and Health Sciences, University of Aden, Al-Jamhoria Hospital, and Ameen Nasher Medical Institute.

### Data management and analysis

Interviews were transcribed verbatim in Arabic and anonymized. Selected quotations were translated into English using a meaning-based approach to preserve conceptual integrity.

Data analysis followed a thematic analytical process informed by Grounded Theory principles and conducted using ATLAS.ti 25. Analysis proceeded through three iterative stages: open coding, axial coding, and selective coding. Open coding was conducted inductively to identify meaning units closely grounded in participants’ accounts. During axial coding, related codes were grouped into categories by examining relationships across governance functions, health system building blocks, and crisis phases. Selective coding integrated these categories into higher-level analytical themes aligned with health system resilience capacities.

Analysis was iterative and comparative, applying constant comparison within and across interviews and across temporal phases. Analytical memos and network visualizations were used to support reflexivity, theory development, and transparency. Data saturation was confirmed when no substantively new themes emerged.

### Trustworthiness and rigor

Rigor was enhanced through prolonged engagement with the data, systematic coding, reflexive memo writing, and temporal comparison across crisis phases. The use of qualitative software supported auditability and consistency, while continuous alignment with established health system and resilience frameworks strengthened analytical credibility.

## Results

### Characteristics of participants

Thirty senior health policymakers and system leaders participated in the study. Participants’ characteristics are shown in Table 1. Most respondents had 20 years or more of professional experience (25/30), and 70% were men. Participants were drawn from multiple departments within the Ministry of Public Health and Population, including the Sectors of Planning, Development and International Cooperation; Primary Health Care; Population and Reproductive Health; Curative and Diagnostic Medicine; and administrative departments affiliated with the Office of the Minister of Health. Five participants represented universities and other health institutions. With respect to temporal coverage, 21 participants reflected primarily on the COVID-19 and post-COVID-19 periods. Five participants provided perspectives spanning all four study periods.

**Table 1:** Background characteristics of participants in the study (*N* = 30)

Characteristics	*n* (%)
Institutional affiliation	
Sector of Planning, Development and International Cooperation (MoPHP)	6(20.0)
Sector of Population and Reproductive Health (MoPHP)	4(13.3)
Sector of Curative and Diagnostic Medicine (MoPHP)	3(10.0)
Sector of Primary Health Care (MoPHP)	6(20.0)
Administrative departments affiliated with the office of the Minister of Health	6(20.0)
Universities and health institutions	5(16.7)
Sex	
Male	21(70.0)
Female	9(30.0)
Years of professional experience	
≥ 20 years	25(83.3)
10–19 years	5(16.7)
Period of involvement	
Pre-2014	2(6.7)
During Conflict (2015–2019)	2(6.7)
During and post COVID	21(70)
All Periods	5(16.6)

### Emerging themes and subthemes

The qualitative analysis identified a set of empirically derived themes and subthemes describing the evolution of health system governance and resilience in Yemen across four distinct periods: pre-2014, conflict escalation (2015–2019), the COVID-19 pandemic, and the post-COVID-19 period. Table 2 summarizes the main themes and subthemes identified across the four periods, along with brief analytic descriptions. The themes map onto key health system resilience capacities-preparedness, absorptive, adaptive, learning/recovery, and transformative capacities. Table 2 provides an overview of the analytical structure, while the sections below elaborate on these findings.

**Table 2:** Thematic analysis of governance and resilience capacities in Yemen’s health system across successive shocks

Period	Main theme	Subtheme	Analytic description
Pre-2014	Foundations without resilience	Centralized governance	Decision-making authority was highly centralized within the MoPHP, limiting sub-national autonomy and constraining local problem-solving and responsiveness. Governance arrangements prioritized administrative control over system learning or risk anticipation.
		Fragile information systems	Health information systems were largely paper-based, fragmented, and underutilized for decision-making, undermining early warning and surveillance capacity.
		Weak emergency preparedness	Despite relative stability, the system lacked structured emergency preparedness plans, contingency financing, and institutionalized risk assessment mechanisms.
		Institutional stagnation	Limited reform momentum, unclear job descriptions, and weak accountability structures constrained institutional learning and adaptability.
2015–2019	Fragmentation and emergency rule	Governance collapse	The outbreak of conflict resulted in fragmented authorities, parallel institutions, and the erosion of formal governance structures, particularly at central level.
		Reactive decision-making	Leadership shifted toward short-term crisis management, with decisions driven by immediacy rather than strategic planning or system strengthening.
		Politicized leadership	Appointments increasingly reflected political affiliation rather than technical competence, weakening institutional legitimacy and continuity.
		Donor-driven survival	Health system functioning became heavily dependent on humanitarian actors, with national leadership assuming a coordinating rather than governing role.
COVID-19	Crisis-driven adaptation	Delayed preparedness	Emergency preparedness mechanisms were activated only after COVID-19 transmission was established, reflecting limited anticipatory capacity.
		Temporary coordination structures	Ad hoc coordination platforms and emergency committees improved cross-actor collaboration but lacked formal mandates or sustainability.
		Non-institutionalized learning	Lessons were identified during the response but were not translated into formal policies, protocols, or structural reforms.
		Missed transformation window	COVID-19 created opportunities for governance reform and decentralization that were not institutionalized once the acute phase subsided.
Post-COVID	Rollback and persistent fragility	Formal preparedness without practice	Preparedness plans exist largely on paper, with limited operationalization, financing, or accountability mechanisms.
		Weak institutional memory	High staff turnover and lack of documentation systems undermined institutional memory and continuity.
		Chronic donor dependence	Continued reliance on external financing constrained national ownership and long-term system transformation.
		Reversion to fragmented governance	Following the COVID-19 period, governance arrangements largely reverted to pre-existing patterns of fragmentation and political interference.

Taken together, the four temporal themes show that Yemen’s health system did not move along a linear pathway from fragility to resilience. Rather, formal governance structures persisted in weakened and changing forms, while the capacities required for resilience remained uneven and poorly institutionalized. Pre-2014 governance provided administrative continuity but limited preparedness. The conflict period shifted governance toward fragmented emergency rule. COVID-19 generated temporary coordination and adaptation, but these gains were not consolidated. In the post-COVID period, formal preparedness and coordination mechanisms remained weakly embedded in routine governance. The central analytical contribution of this study is therefore to show how health system governance can remain institutionally visible while failing to generate learning and transformative resilience.

### Pre-2014: Foundations without resilience

Participants consistently described the pre-2014 period as one of relative administrative stability but limited institutionalized resilience. Governance within the MoPHP was characterized by centralized authority, rigid planning processes, weak risk anticipation, and limited institutional learning. Although formal structures and policies existed, they functioned largely as administrative instruments and did not translate into operational preparedness or adaptive capacity.

#### Centralized governance and constrained local responsiveness

The narrative showed that decision-making authority prior to 2014 was highly centralized at the national level, limiting flexibility and problem-solving capacity at governorate and district levels. Subnational health offices were largely confined to implementation roles, with minimal involvement in planning or situational analysis. This governance model positioned local actors as executors rather than decision-makers, constraining responsiveness to contextual variation.

As one senior policymaker explained:

*One of the fundamental problems of Yemen’s health system prior to 2014 was that national health plans were largely confined to the Ministry of Public Health and Population and a small circle of advisors*,* while governorate and district health offices were relegated to implementation roles without meaningful involvement in decision-making*,* planning*,* or situational analysis.* (KI-29; Female, Professional Experience 15 years)

Several respondents recalled that due to this centralized governance system, even minor operational adjustments required clearance from the central level, slowing response times and discouraging initiative. Over time, this produced a culture of administrative compliance rather than proactive problem-solving. As one long-serving official noted:

*Everything had to go back to the center. Even small adjustments needed approval. Over time*,* this discouraged local teams from taking responsibility or proposing solutions.* (KI-5; Female, Professional Experience 25 years)

Participants further emphasized that the centralized governance shaped uneven system performance, with infrastructure, training opportunities, and specialized services disproportionately concentrated in Sana’a. Governorates with fewer political or administrative ties to the center reported persistent gaps in service availability and managerial support.

One respondent described how political and administrative centralization translated into material disparities:

*Before 2014*,* health system centralization was heavily skewed toward Sana’a. Advanced laboratory services-including PCR [Polymerase Chain Reaction] testing and diagnostics for influenza and measles-were almost exclusively available in Sana’a*,* despite Aden hosting a central public health laboratory formally affiliated with the Ministry of Public Health. Capacity building*,* equipment*,* and technical development were largely concentrated in the capital.* (KI-29; Female, Professional Experience 15 years)

This was supported by another professional with over 25 years of professional experience:

*Prior to 2014*,* most resources were directed to major hospitals in Sana’a. This resulted in marked disparities not only in infrastructure*,* but also in service quality*,* equipment*,* staff training*,* infection control*,* bed capacity*,* specialized ICUs [Intensive Care Units]*,* and accredited training centers.* (KI-30; Male, Professional Experience 25 years)

Although intersectoral coordination mechanisms formally existed, participants described them as reactive and event-driven rather than embedded in routine governance processes. This limited the system’s ability to anticipate risks or coordinate preventive action, reinforcing a pattern in which stability masked underlying fragility.

#### Weak information systems and surveillance capacity

Health information systems during this period were described as fragmented, predominantly paper-based, and underutilized for decision-making. Except for immunization-related systems-supported by sustained financing and technical leadership-most programs lacked reliable data flows. One public health expert noted:

*Prior to 2014*,* health information systems were predominantly paper-based and lacked the capacity to effectively inform decision-making. There were no functional information systems for nutrition or reproductive health*,* while immunization-related information systems were the only operational and reliable systems.* (KI-4; Male, Professional Experience 40 years)

Additionally, participants revealed that disease surveillance and early warning mechanisms were weak, non-systematic, and not institutionalized and relied heavily on individual initiative. The absence of systematic surveillance undermined early detection and response to outbreaks and limited the health system’s ability to learn from recurring public health threats. Reflecting on epidemic preparedness, participants had this to say:

*There was no clear mechanism for epidemic surveillance. Surveillance relied on the individual efforts of those working in the system. Despite these efforts*,* the response was fragmented. Essentially*,* the system did not truly exist in an institutional sense*. (KI-30; Male, Professional Experience 25 years)

*The surveillance system was weak even before 2014*,* even in terms of response. Sometimes information about outbreaks reached the media before it reached the health system.* (KI-4; Male, Professional Experience 40 years)

#### Formal planning without preparedness or learning

Although annual plans and sectoral strategies existed, participants described these as routine administrative exercises rather than tools for anticipating shocks. Emergency preparedness was largely absent, reflecting a broader lack of risk-oriented governance.

A policymaker summarized:

*We had plans*,* but they were normal plans. There was no thinking about emergencies or shocks. No preparedness mindset at all.* (KI-3; Male, Professional Experience 20 years)

Institutional learning mechanisms were similarly weak. Despite repeated exposure to smaller-scale emergencies, lessons were not systematically captured or translated into preparedness planning. As a result, the system entered the conflict period without accumulated institutional memory or adaptive routines. A participant lamented:

*Despite repeated epidemics since 2010*,* including dengue and H1N1 influenza*,* there is no preparedness planning or systematic learning from prior outbreak responses.* (KI-29; Female, Professional Experience 15 years)

#### Institutional stagnation and political interference

Participants emphasized that transformative capacity before 2014 was minimal. Governance reforms were limited, job descriptions for leadership positions were largely absent, and accountability mechanisms were weak.

*At the level of the Ministry of Public Health and Population*,* there are no formal job descriptions for any key leadership positions… Personally*,* despite working in the health sector for over twenty years*,* I have never received a formal job description.* (KI-15; Male, Professional Experience 20 years)

Political interference and outdated legislation further constrained institutional modernization.

### Conflict period (2015–2019): fragmentation and emergency rule

The outbreak of armed conflict in 2015 marked a structural rupture in Yemen’s health system. As reflected in Table 2, this period was characterized by governance collapse, reactive decision-making, politicized leadership, and donor-driven survival strategies. While health services continued to function under extreme constraints, system survival relied increasingly on ad hoc arrangements and external actors rather than coherent state-led governance.

#### Collapse of governance and parallel authorities

Participants consistently described the early conflict period as one of institutional fragmentation. The division of political authority resulted in parallel ministries, overlapping mandates, and unclear lines of accountability. Central governance capacity deteriorated rapidly, and strategic stewardship was displaced by ad hoc crisis management.

One respondent captured this shift succinctly:

*Between 2015 and 2019*,* the Ministry of Public Health was effectively split into two parallel ministries. This severely disrupted functionality*,* weakened accountability*,* and made meaningful professional engagement untenable.* (KI-2; Male, Professional Experience 20 years)

A respondent added:

*Due to the existence of two authorities-a legitimate government and an insurgent authority-the capital was moved to Aden*,* requiring the rebuilding of institutions. The legitimate government operated from outside the country.* (KI-28; Male, Professional Experience 30 years)

The results also revealed that the scale and depth of institutional collapse were unexpected, further exposing the absence of preparedness:

*There was no anticipation that things would reach this level. No one expected the collapse to be so deep.* (KI-1; Female, Professional Experience 25 years)

#### Reactive leadership and politicized appointments

Despite early recognition that the conflict may prolong, preparedness planning remained absent. Leadership practices became increasingly reactive, driven by immediate pressures rather than long-term strategy. Participants emphasized that leadership effectiveness depended heavily on individual competence rather than institutional safeguards.

As one participant noted:

*If a director was strong*,* the facility worked. If not*,* everything collapsed. There was no system to absorb pressure.* (KI-12; Female, Professional Experience 30 years)

Political affiliation increasingly influenced appointments to leadership positions, eroding professional legitimacy and institutional trust.

*Appointments were often made deliberately to individuals without qualifications so they could be easily controlled. Those who were qualified tended to leave quickly because they would not accept wrongdoing.* (KI-29; Female, Professional Experience 15 years)

Frequent leadership turnover further weakened continuity and learning as a participant describes:

*After the 2014 war*,* new leadership figures emerged-people who had not previously been part of the system-who suddenly assumed responsibilities without having sufficient professional background or experience*. (KI-1; Female, Professional Experience 25 years)

#### Donor-driven survival and erosion of national ownership

During this period, the health system’s ability to continue functioning relied heavily on humanitarian financing and international actors. Donors and UN (United Nations) agencies assumed leading roles in service delivery, coordination, and planning, while national authorities shifted toward a facilitative role.

One senior policymaker explained:

*Humanitarian emergency response planning and inter-sectoral coordination were largely led by OCHA [United Nations Office for the Coordination of Humanitarian Affairs]*,* with health interventions coordinated between donors and international organizations through this mechanism.* (KI-2; Male, Professional Experience 20 years)

While this external support enabled short-term absorption of shocks, participants emphasized that it reinforced parallel coordination structures and constrained opportunities for institutional strengthening and national ownership.

*Following 2014*,* Yemen’s health system became heavily dependent on international aid to cover its basic operational needs. This dependency undermined autonomous planning*,* weakened governance structures*,* and increased the influence of international organizations over national priorities.* (KI-4; Male, Professional Experience 40 years)

#### Absorptive response without institutional learning

Although the conflict generated substantial operational experience, participants reported that learning remained fragmented and individual rather than institutional. Risk assessments, after-action reviews, and governance reforms were largely absent, reinforcing a cycle of reactive response and repeated failure. A public health expert explained:

*The crises occurring between 2014 and 2017 were met without a clear institutional response. No formal risk assessments were conducted*,* and no systematic lessons were documented or integrated into planning*. (KI-5; Female, Professional Experience 25 years)

This pattern was further reinforced by the absence of system-level reflection following response efforts:

*We responded*,* yes*,* but we did not sit as a system to evaluate what went wrong and how to fix it. Learning remained individual and fragmented rather than institutional.”* (KI-4; Male, Professional Experience 40 years)

### COVID-19 period: Crisis-driven adaptation and a missed transformation

The COVID-19 pandemic represented a system-wide shock that temporarily altered governance practices and decision-making dynamics within Yemen’s health system. Participants described this period as one of crisis-driven adaptation, marked by delayed preparedness, temporary coordination gains, and non-institutionalized learning.

#### Delayed preparedness and fragmented activation

Participants unanimously reported that Yemen entered the COVID-19 pandemic without operational readiness. Preparedness efforts were activated only after the confirmation of cases, reflecting limited anticipatory capacity and unclear leadership mandates.

As one participant reflected:

*We did not prepare because COVID-19 was coming; we prepared because it had already arrived.* (KI-28; Male, Professional Experience 30 years)

Some participants also highlighted that uncertainty over institutional authority and fear of political repercussions may have further delayed early action, reinforcing a pattern of reactive rather than preventive response.

#### Temporary coordination and absorptive gains

COVID-19 prompted the rapid establishment of emergency committees, task forces, and centralized coordination mechanisms, and clearer leadership hierarchies. Decision-making accelerated, and some decentralization occurred, allowing governorates greater flexibility. However, the participants acknowledged that these arrangements improved absorptive capacity but were explicitly temporary and crisis driven rather than structured reforms.

A senior official noted:

*During COVID-19*,* decisions moved faster-but only because everything was treated as exceptional.* (KI-20; Female, Professional Experience 15 years)

#### Informal adaptation and workaround governance

Adaptive responses during COVID-19 relied heavily on improvisation, including informal practices. Isolation centers were established rapidly, oxygen supply infrastructure expanded, and service delivery models modified. Informal digital platforms-particularly WhatsApp-emerged as key coordination tools in the absence of functional information systems.

One participant explained:

*WhatsApp became our situation room. Decisions*,* data*,* everything went through it.* (KI-12; Female, Professional Experience 30 years)

While effective in the short term, these workarounds lacked formal mandates, documentation, and accountability.

#### Non-institutionalized learning and missed reform opportunity

Although evaluations and reflections occurred, participants emphasized that learning remained donor-driven and project-specific. Lessons were not translated into revised policies, financing mechanisms, or governance reforms.

As one policymaker summarized:

*COVID-19 showed us what was possible*,* but once the crisis passed*,* the system went back to normal.* (KI-21; Male, Professional Experience 25 years)

## Post-COVID-19 period: Rollback and persistent fragility

The participants described the post-COVID-19 period as a period characterized by a rollback of emergency gains and the re-emergence of pre-existing governance weaknesses (Table 2). Temporary coordination gains and adaptive practices introduced during the pandemic were not sustained, and the health system largely reverted to fragmented and reactive modes of operation.

### .

#### Formal preparedness without operational readiness

Participants reported that preparedness plans and risk assessments now formally exist within the health system; however, these instruments were widely described as procedural rather than operational. Respondents emphasized that preparedness has not been translated into concrete readiness measures, such as pre-positioned resources, dedicated contingency financing, clear command structures, or routinely tested response protocols. As a result, emergency plans remain disconnected from day-to-day system functioning and are rarely activated until a crisis is already underway. One respondent summarized this gap between formal preparedness and practical readiness by noting that preparedness remains largely symbolic, with each new shock triggering an improvised response rather than the activation of established systems:

*Preparedness exists on paper. When a shock happens*,* we start from zero.* (KI-5; Female, Professional Experience 25 years)

#### Declining absorptive capacity and donor dependence

As emergency funding associated with COVID-19 declined, participants reported a sharp deterioration in absorptive capacity. Facilities struggled to maintain essential services, and emergency buffers established during the pandemic were not institutionalized. One senior policymaker explained:

*During COVID-19 we could absorb the shock because funds were available. Now even small outbreaks strain the system.* (KI-30; Male, Professional Experience 25 years)

Respondents consistently highlighted continued reliance on external financing and humanitarian actors, noting that donor priorities continued to shape service delivery and coordination arrangements, often at the expense of long-term system strengthening.

#### Weak institutional memory and accountability

Participants emphasized that institutional memory remained fragile in the post-COVID-19 period. High staff turnover, fragmented documentation, and donor-driven evaluations undermined institutional memory. Lessons from COVID-19 were not embedded into governance frameworks or accountability mechanisms. Moreover, although reports and evaluations were produced, they were rarely used to inform governance reform or planning.

A participant reflected:

*Reports were written*,* but no one asked: what did we learn*,* and what should change? *(KI-4; Male, Professional Experience 40 years)

#### Reversion to fragmented governance and stalled transformation

Finally, participants described a clear return to fragmented authority, political interference, and weak intersectoral coordination. Transformative reforms initiated during the pandemic such as accelerated decision-making and temporary decentralization, were not sustained.

A participant concluded:

*This was not a period of transformation. It was a period of survival.* (KI-29; Female, Professional Experience 15 years)

## Discussion

This study provides a longitudinal governance-focused analysis of health system resilience in Yemen across four major shock periods. Drawing on senior policymakers’ perspectives, the findings show that while formal governance structures persisted throughout the past decade, they were not accompanied by the institutional capacities required to anticipate, absorb, adapt to, learn from, or transform in response to repeated crises. From a resilience perspective, Yemen’s health system exhibits a pattern of procedural preparedness, externally buffered absorption, reactive and localized adaptation, fragmented learning, and limited transformative change. This trajectory aligns with resilience literature emphasizing that resilience is not synonymous with crisis response or service continuity but depends on the ability to reconfigure governance arrangements and institutional rules under sustained stress [2, 13]. The discussion below examines these findings using a sequential resilience framework-preparedness, absorption, adaptation, learning/recovery, and transformation capacities-tracing how each evolved across the four phases and how governance arrangements shaped their expression.

The findings also show that governance failure in Yemen was not only administrative or technical but political. Decision-making authority became fragmented across competing institutional centres; donor financing increasingly substituted for domestic fiscal authority; and accountability was weakened by divided mandates, unstable leadership, and limited enforcement capacity. This political economy helps explain why formal governance structures could persist while resilience capacities remained weak: institutions continued to exist, but the authority, financing, legitimacy, and learning mechanisms required to convert crisis response into system transformation were not consolidated.

### Preparedness capacity: from administrative stability to procedural readiness

Prior to 2014, preparedness was constrained by centralized governance, rigid planning processes, and weak risk anticipation. Although formal plans existed, they functioned largely as administrative artifacts rather than operational tools. Subnational actors had limited authority, undermining local situational awareness and responsiveness. These findings are consistent with evidence that preparedness requires decentralized decision-making, embedded surveillance, and scenario-based planning rather than static documentation [1, 18, 19].

The conflict period exposed and amplified these weaknesses. Fragmented authority and emergency governance displaced strategic planning and accountability, reinforcing short-term survival approaches typical of fragile and conflict-affected settings [12, 20, 21]. The cholera epidemic exemplified how weak preparedness and coordination constrained effective multisectoral response [7, 22, 23].

COVID-19 temporarily mobilized preparedness mechanisms, but activation remained delayed and poorly institutionalized. As in other politically fragmented settings, formal preparedness frameworks proved insufficient without clear mandates and enforcement [24, 25]. Post-pandemic, preparedness largely reverted to a procedural state, reinforcing critiques that preparedness remains symbolic unless embedded as a core governance function with dedicated financing and authority [19, 26].

### Absorptive capacity: continuity through external buffering rather than internal resilience

Across all phases, absorptive capacity depended primarily on humanitarian financing and donor-led mechanisms rather than domestic buffers. During the conflict, continuity of services relied on external substitution, with national authorities assuming a coordinating role. This mirrors evidence from Fragile and Conflict-Affected Situations (FCAS) where donor dependence sustains service delivery but erodes stewardship and accountability [12, 20].

COVID-19 temporarily strengthened absorptive capacity through emergency financing and centralized procurement, but these gains were not sustained. As donor funding contracted post-pandemic, absorptive capacity deteriorated sharply, highlighting the vulnerability of systems lacking domestic contingency financing and fiscal space [21, 25].

### Adaptive capacity: reactive improvisation and uneven decentralization

Adaptive responses were pragmatic but weakly institutionalized, producing uneven performance across regions. During conflict, adaptation relied heavily on individual leadership and informal practices-forms of “everyday resilience” that enabled short-term continuity but concealed systemic fragility [27].

COVID-19 accelerated adaptation through task-shifting, service reconfiguration, and informal digital coordination. The emergence of WhatsApp-based governance illustrates how informal tools can temporarily compensate for weak systems, a pattern documented in other crisis contexts [28, 29]. However, without formal integration, such adaptations remained reversible. Post-COVID, most initiatives were not embedded into routine governance, reinforcing evidence that adaptation without codification rarely endures [11].

### Learning and recovery: experience without institutional memory

Learning capacity is critical for converting adaptation into transformation and depends on structured reflection, feedback mechanisms, and institutional memory [19, 26, 30].Across all phases, learning in Yemen was described as fragmented, donor-driven, and project-specific.

Although valuable experience accumulated during conflict and COVID-19, lessons were rarely institutionalized. Evaluations were typically externally driven and weakly connected to governance reform, reflecting broader evidence from FCAS where unstable leadership, parallel reporting systems, and fragmented data ownership undermine learning [12, 20]. WHO guidance emphasizes the role of after-action reviews in fostering reform, yet their impact depends on political commitment and institutional ownership-both limited in Yemen [30, 31].

Post-COVID, learning remained weak, allowing governance failures to recur across successive shocks. Participants noted that reports were produced without corrective action, reinforcing findings that learning requires accountability mechanisms linking evidence to decision-making and resource allocation [18, 19, 26].

### Transformative capacity: missed windows and reversion to equilibrium

Despite extensive crisis experience, learning remained fragmented and donor-driven. Evaluations were weakly connected to governance reform, reflecting broader challenges in FCAS where parallel reporting systems and unstable leadership undermine institutional memory [20]. The absence of accountability mechanisms linking evidence to decision-making limited learning and enabled repeated failures across shocks [19].

Transformative capacity was largely absent. Although COVID-19 created a temporary policy window, reforms were not sustained once the emergency phase passed. This reversion underscores a central insight from resilience scholarship: without embedding crisis-driven changes into routine governance, financing, and authority structures, systems revert to prior equilibria [11, 26].

This study offers a rare longitudinal qualitative analysis of health system governance in Yemen across four distinct periods of shock. Drawing on insights from experienced informants at leadership and technical levels, it provides theory-informed understanding of how governance arrangements shape preparedness, absorption, adaptation, learning, and transformation over time. The resilience-capacity framework enhances analytical clarity and strengthens the study’s relevance for policy development and resilience programming in fragile and conflict-affected settings.

Several limitations merit consideration. First, the study relies on retrospective accounts, which may be subject to recall bias, particularly regarding the pre-2014 and early conflict periods. Second, as is common in governance research in fragile and conflict-affected settings, social desirability bias may have shaped narratives around political interference, accountability, and institutional performance. Third, although the sample includes diverse information, the findings may not capture all perspectives across governorates or among actors outside formal institutions. Fourth, Yemen’s fragmented and fluid governance environment means that some views may reflect localized experiences rather than nationally consistent patterns. Nonetheless, triangulation across interviews and the repeated convergence of themes support the credibility of the findings.

### Policy implications for Yemen and other fragile settings

These findings suggest that strengthening resilience in Yemen requires moving from ad hoc crisis coordination to institutionalized governance mechanisms. First, emergency committees and coordination platforms should be embedded within routine governance structures with clear mandates, decision rights, and accountability procedures. Second, emergency preparedness should be supported by protected contingency financing and recurrent operations and maintenance budgets, rather than relying primarily on short-term donor surges. Third, adaptive practices that emerged during COVID-19, such as rapid coordination, task reallocation, and informal digital communication, should be formalized where appropriate through protocols, supervision systems, and integrated health information platforms. Fourth, after-action reviews and learning mechanisms should be linked to budgetary decisions, workforce planning, and regulatory reform so that lessons from shocks become enforceable institutional change rather than project-specific documentation.

Although Yemen’s political fragmentation is context-specific, the pattern observed here is relevant to other fragile and conflict-affected settings: formal institutions may continue to operate while the capacities needed for preparedness, absorption, adaptation, learning, and transformation remain weak. For such settings, resilience programming should therefore assess not only whether governance structures exist, but whether they have the authority, financing, information systems, and accountability mechanisms required to translate crisis experience into durable institutional reform.

## Conclusion

This study suggests that the persistence of formal health governance structures does not necessarily translate into health system resilience. Across successive shocks in Yemen, formal institutions, committees, and coordination mechanisms continued to exist, but preparedness remained weak, absorptive capacity depended largely on external humanitarian support, adaptation was often temporary and localized, and learning rarely became institutionalized reform. Before 2014, governance was administratively stable but centralized and weakly oriented towards risk and preparedness. During the conflict, institutional fragmentation and emergency rule displaced strategic stewardship. COVID-19 temporarily strengthened coordination and operational adaptation, but these gains were not sustained. In the post-COVID period, declining donor support, weak enforcement, and unresolved political fragmentation have continued to constrain the transition from crisis coping to transformative resilience.

For Yemen and other fragile and conflict-affected settings, strengthening resilience requires more than preserving formal governance structures or mobilizing short-term emergency responses. It requires clearer decision rights, accountable mandates, protected contingency and recurrent financing, workforce mechanisms, integrated information systems, and routine learning processes that can persist between shocks. Progress should therefore be judged not only by whether the health system survives the next crisis, but by whether each crisis leaves the system more coherent, accountable, and capable of institutional transformation.

## Supplementary Information

Below is the link to the electronic supplementary material.

Supplementary Material 1

## Data Availability

The datasets used and/or analyzed during the current study are available from the corresponding author upon reasonable request.

## Abbreviations

FCAS: Fragile and Conflict-Affected Situations
ICU: Intensive Care Unit
MoPHP: Ministry of Public Health and Population
OCHA: United Nations Office for the Coordination of Humanitarian Affairs
PCR: Polymerase Chain Reaction
UN: United Nations
WHO: World Health Organization
